# Relationships between magnitude representation, counting and memory in 4- to 7-year-old children: A developmental study

**DOI:** 10.1186/1744-9081-6-13

**Published:** 2010-02-18

**Authors:** Fruzsina Soltész, Dénes Szűcs, Lívia Szűcs

**Affiliations:** 1 Centre for Neuroscience in Education, Faculty of Education, University of Cambridge, UK; 2 Research Institute for Psychology, Hungarian Academy of Sciences, Budapest, Hungary; 3 Kikelet Óvoda, Nyíregyháza, Hungary

## Abstract

**Background:**

The development of an evolutionarily grounded analogue magnitude representation linked to the parietal lobes is frequently thought to be a major factor in the arithmetic development of humans. We investigated the relationship between counting and the development of magnitude representation in children, assessing also children's knowledge of number symbols, their arithmetic fact retrieval, their verbal skills, and their numerical and verbal short-term memory.

**Methods:**

The magnitude representation was tested by a non-symbolic magnitude comparison task. We have perfected previous experimental designs measuring magnitude discrimination skills in 65 children kindergarten (4-7-year-olds) by controlling for several variables which were not controlled for in previous similar research. We also used a large number of trials which allowed for running a full factorial ANOVA including all relevant factors. Tests of verbal counting, of short term memory, of number knowledge, of problem solving abilities and of verbal fluency were administered and correlated with performance in the magnitude comparison task.

**Results and discussion:**

Verbal counting knowledge and performance on simple arithmetic tests did not correlate with non-symbolic magnitude comparison at any age. Older children performed successfully on the number comparison task, showing behavioural patterns consistent with an analogue magnitude representation. In contrast, 4-year-olds were unable to discriminate number independently of task-irrelevant perceptual variables. Sensitivity to irrelevant perceptual features of the magnitude discrimination task was also affected by age, and correlated with memory, suggesting that more general cognitive abilities may play a role in performance in magnitude comparison tasks.

**Conclusion:**

We conclude that young children are not able to discriminate numerical magnitudes when co-varying physical magnitudes are methodically pitted against number. We propose, along with others, that a rather domain general magnitude representation provides the later basis for a specialized representation of numerical magnitudes. For this representational specialization, the acquisition of the concept of abstract numbers, together with the development of other cognitive abilities, is indispensable.

## Background

It is a major question whether the representation of approximate numerical magnitudes in children develops and sharpens independently of symbolic arithmetical abilities, or symbolic knowledge correlates with the approximate magnitude representation in some ways. There is a sharp divide in the corresponding developmental literature: many argue that the innate analog magnitude representation is a prerequisite of the acquisition of arithmetics; others claim that formal education and numerical enculturation sharpens the analog magnitude representation in children. On the one hand, several researchers assume that children have an innate, preverbal approximate, language-independent magnitude representation shared with other species [1-11]. According to this account, refinement of the analogue magnitude representation correlates with math achievement [12-14] and has a predictive value for later math performance [15,16]. On the other hand, others think that the relation is reversed. Development and sharpening of magnitude representation is supported by language, especially by counting skills [17-19].

Number representation skills are most frequently tested by quantity discrimination tasks (or by number line estimation [12]; however magnitude discrimination and estimation are strongly interrelated [20,21]). In these tasks, infants are expected (and older children are explicitly asked) to discriminate between perceptual displays showing a certain number of items (e.g. dots). The most general finding is that quantity discrimination depends on the ratio of to-be-compared quantities. It is harder to compare quantities when their ratio is closer to 1 than when their ratio is further away from 1. The ratio effect has been consistently shown in infants (symbolic stimuli: [22,23]; non-symbolic stimuli: [4,5,10,11,16,18,24-30]), and also in animals and human adults. Hence, it is thought that numbers are coded in analogous, approximate fashion by an evolutionarily grounded pre-verbal magnitude representation [31-34].

The most important methodological challenge in magnitude discrimination experiments is that perceptual variables are inevitably correlated with number. These variables correlate both with each other and with numerosity and it is impossible to control for all of them at the same time. For instance, if intensive properties (individual item properties, like item size) are kept equal in a particular trial, extensive properties (properties of the set, like summed surface of all items in a group) will inevitably co-vary with number, and vice versa. With a simple example, a collection of 6 apples is not only more, but physically also larger than a collection of 3 apples. In nature, 'more' usually correlates with 'bigger' (number of individuals in a group, number of pieces of food, etc.). Infants can rely on these simple perceptual features of sets, instead of the more abstract property of numerosity. Several of the early studies did not control for these perceptual correlates of the stimuli [26,27] making infants' putative numerical performance indistinguishable from their perceptual performance. In fact, when overall surface [35] or circumference [36,37] is controlled during experiments, infants are more sensitive to the continuous perceptual variable than to number. It was also shown that infants habituated to total surface area but not to number when these two dimensions were pitted against each other, i.e. when the numerically 'more' set was smaller in physical size [38].

Xu and Spelke [4] devised a habituation paradigm in which they attempted to control for the non-numerical perceptual variables. They varied sum surface and density of the trials in a way that nothing but the number changed from habituation to test trials. They showed that 6-month-old infants were sensitive to number change, independent of perceptual variables. These results have been replicated and extended by several later experiments, leading to the conclusion that infants possess a basic understanding and representation of approximate numbers [for a review, see [9]], providing the basis for the acquisition of later arithmetics.

Contrary to the above statement, other researchers who investigated the co-development of the number representation and verbal counting skills in young children, arrived to the conclusion that verbal counting knowledge is inevitable for the abstraction of numerical magnitudes [5,17-19,39-42]. For example, Mix and colleagues [39,41,42] found that 3-4-year-old children could not match cross-modal stimuli based on numerosity before they were able to master the verbal counting system. Brannon and Van de Walle [17] and Rousselle et al [18] also found that only children who already mastered and understood the verbal counting system and were able to use the role of cardinality, were able to discriminate numerical magnitudes independent of their perceptual properties, like overall size. However, the relationship between number discrimination and verbal counting knowledge disappears after the very first stages of the acquisition of the latter. This suggests that the acquisition of verbal counting abilities enables children to understand that numerical quantities are independent of objects' physical properties, like size and luminance. Children who have not yet experienced this conceptual shift do not understand the abstract nature of numbers and rely on analogue perceptual features in number comparison tasks [17,18].

The inability of 3-4 year-old children to avoid the effect of perceptual variables apparently contradicts findings according to which even infants are able to discriminate dot patters based purely on their numerosity when perceptual variables are controlled for [e.g., [4,38,43]]. However, there is a perceptual confound still unaccounted for in Xu and Spelke's [4] paradigm. Controlling, i.e. keeping constant overall surface, will cause item size to covary with number. In fact, the distribution of item sizes across trials is very different from the distribution of sum surface across trials and it is correlated with the numerosity of the dots. More precisely, and according to the authors' stimuli description, the diameter of an item varies between 1.06-2.37 cm in the 8-item displays and between 0.75-1.67 cm in the 16-item displays. As item diameter on test displays is 1.5 cm, item size of dots in an 8-item test display is larger than the average item size in a 16-item habituation display (1.3). It is possible that infants reacted to the change in dot size, instead of the number of the dots.

Here, we set out to explore the developmental relations between magnitude representation, number knowledge and counting skills in preschool children, using an improved number comparison paradigm and also measuring reaction time in addition to accuracy. We utilized a number discrimination paradigm similar to the one used by Rousselle and Noël [19]. They not only equated some perceptual variables across trials, but in some instances, pitted perceptual properties against number. In one third of the trials, number and physical properties were congruent: the numerically larger set was physically larger as well. Another third of the trials were incongruent: the numerically larger set was smaller in physical size. The last third of trials were neutral: sum surface was equated among the dot sets. We consider the manipulation of congruency as the most optimal way to control for perceptual properties. As the equation of any of the perceptual properties (e.g. sum surface) yields that another property (e.g. item size) will correlate with number, probably the best solution is to explicitly oppose the physical and numerical dimensions. In the incongruent situations, most of the perceptual variables will be the opposite of the numerical property: density, item size, sum surface, sum circumference etc. will all be smaller in the numerically larger set. Meanwhile, in the congruent situations item size, sum surface, density etc. will all be larger in the numerically larger set. If children were relying on any of the perceptual properties, these properties would lead to the incorrect answer in a significant portion of the trials.

The co-development of the number representation and verbal counting skills is mostly measured by correlating performance on a non-symbolic magnitude comparison task and the performance on some verbal tasks. We used the most commonly used verbal counting measures, the 'how many' task, the 'give a number' task and the 'how high' task, measuring the understanding of one-to-one correspondence, counting and cardinality [8,18,44].

Further, we added control measures of verbal fluency, short term memory for numbers, short-term memory for words, arithmetic problem solving (thought to be based on memory retrieval), line halving and number knowledge, in addition to measuring counting abilities. We were motivated by a growing literature emphasizing the role of memory in the aetiology of numerical disabilities [e.g., [45-47]]. For example, children with mathematical disabilities have difficulties in rehearsing verbal information and in control processes attributed to the central executive [48]. They also have verbal fluency difficulties [45,49,50]. Memory and control processes are also important in normal numerical development [[51,52]; however, see [53,54] for an opposite opinion].

We also aimed to identify the relevant and possibly interrelated developmental factors behind number discrimination performance and counting knowledge. We were interested whether the ability to judge pairs of sets based on numerosity, independently of the competing perceptual information, would or would not correlate with children's verbal counting knowledge. Based on the literature, we expect that number discrimination performance and verbal counting knowledge are independent of each other after three years of age [e.g., [17,18]]. Further, we expect to find a developmental change across age groups in the ability to resist task-irrelevant and conflicting perceptual information and in the ability to discriminate close magnitudes. These developmental factors probably reflect the maturation of more general abilities (i.e. executive functioning, attention and memory, for an overview see [55]).

We predict that the congruency effect will weaken by age because inhibitory control substantially develops in children during the age range examined. A ratio effect is also expected, reflecting the approximate nature of magnitude representation. The most interesting question is whether counting/number knowledge and markers of the magnitude representation, i.e. the ratio effect, correlate with each other. One possibility is that there is such correlation. This would support that 1) either the accuracy of the non-symbolic magnitude representation predicts arithmetic performance [e.g., [16]], or 2) that verbal counting knowledge supports non-symbolic number representation [17,18]. Another possibility there is no such correlation. This would suggest that counting abilities and number knowledge follows a developmental track independent of that of non-symbolic magnitude comparison and the two form two independent developmental factors.

## Methods

### Subjects

65 children attending a public kindergarten participated in the experiment which was carried out in Hungary (Nyíregyháza). Children came from a working- or middle-class background. Children assigned to different age groups entered kindergarten in consecutive years. There were 14 4-year olds (7 boys, mean = 4 years, SD = 0.14 years), 17 5-year-olds (7 boys, mean = 5.6 years, SD = 0.28 years), 17 6-year-olds (8 boys, mean = 5.96, SD = 0.24) and 17 7-year-olds (9 boys, mean = 6.88 SD = 0.28). Written informed consent was obtained from parents and the study was approved by the institutional ethics committee of the Research Institute for Psychology at the Hungarian Academy of Sciences.

### Procedure

Data were collected in two sessions. During the first session behavioural tests were administered. During the second session the computerized magnitude comparison test was administered.

### Tests

There were twelve behavioural tasks. The tasks were grouped into thematic sets for presentation purposes. The order of sets was counterbalanced during administration in order to avoid systematic effects arising from general fatigue or boredom. The first thematic set consisted of two tests measuring children's *number knowledge: *"Say as many numbers as you can" (number recitation) and a number recognition task (Arabic numerals from 1 to 10 were shown in random order). Children were scored for each number they said and for each Arabic numeral they named correctly. The second set of two tests measured *verbal fluency*, including two common tasks in categories which are familiar to young children [for example, [56]]: "Say as many animals as you can" and "Say as many colours as you can". Children were given a score for every word that they generated in one minute. The third set of two tasks assessed children's *verbal short term memory*: auditory short term memory for numbers and auditory short term memory for words. Children's score was the largest number of items they recited correctly. The fourth set contained three tasks measuring *counting abilities: *"Count as far as you can" (knowledge of the verbal counting system), "How many toys are there" (one-to-one correspondence and cardinality) and "Give me a number" (counting) tasks. These measures for assessing children's counting abilities followed the design of Wynn [8,44] and Rousselle et al. [18]. Children were scored on the "Count as far as you can" task according to the maximum number that they could count to without committing an interchange or an omission. The "How many toys are there" task score reflected the maximum number of elements (toy cars) children could count correctly. The "Give me a number" task score was determined by the maximum number of toys children could select correctly according to the instruction. The number of elements in "How many toys are there" and "Give me a number" was extended (to maximum 100) until two consecutive errors were made by the child. After presenting/asking for 2-5 items, item number was randomly extended (e.g. 8, 13, 15, 20). The fifth set consisted of two tasks measuring *verbal problem solving abilities*: easy problems and difficult problems. Easy problems were additions with the same numbers, e.g. 1+1, 2+2; these additions often come up in kindergarten activities (6 problems; following the curriculum in Hungarian kindergartens). Here the correct answer could be simply recited from memory (as children have already overlearned them in short rhymes). More difficult problems could not be easily recited from memory. These problems consisted of additions using the same numbers as the easy problems but were not overlearned by children (15 problems, e.g. 2+5). The problems were presented verbally and had to be answered verbally. Children were scored according to the number of additions that they could solve. The difficulty of additions was also increased by increasing the problem size (e.g. the simplest problem was 1+1, then 2+2, then 3+3 and so on). The seventh measure characterized children's understanding of halving and their estimation abilities. According to the literature, estimation also reflects the magnitude representation and correlates with performance on magnitude discrimination tasks [20,21]. In this task children were asked to share a salty stick (approximately 13 cm in real life) with their peer equally. A drawing of ten sticks (10 to 20 cm) was shown and they were asked to mark the point where they would break the real stick to share it equally. Performance was measured in terms of deviation in millimetres from the middle point.

### Magnitude comparison task

**Figure 1:**
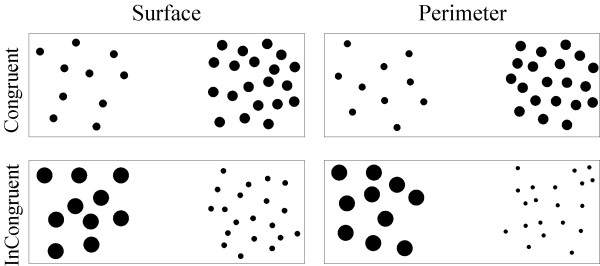
**Example stimuli**.

The thirteenth task was the magnitude comparison task. Black dots on a light yellow background were used as stimuli. Two sets of dots were presented simultaneously on a computer screen (see Figure 1).

We used only congruent and incongruent trials, so that perceptual features were pitted against number in exactly 50% of the trials. We omitted the neutral situation because neutral trials always contain at least one physical feature which is correlated with number (for example, density provided a reliable cue for numerosity both in the congruent and in the neutral trials in the Rouselle and Noël [19] study). Congruent and incongruent trials were intermixed with each other. In consequence, attending to a certain perceptual cue would lead systematically to the correct answer in the congruent condition and to the incorrect answer in the incongruent condition. By having incongruent condition, we do not need to worry about all the perceptual variables which are impossible to control for at the same time: when for example the sum surface is incongruent with number, item size, or density are also incongruent with number. We also controlled for two perceptual features at the same time, so that both perceptual controls were included in the same experiment, intermingled in a pseudo-random way within each block and within each subject. We chose to control for overall surface area and circumference, because these were found to be more salient features than number for children of this age (surface: [18,38,57]; circumference: [36]). We were interested if either of these perceptual controls were more salient to children. The two different types of perceptual control are also shown in Figure 1.

The sets were separated by 7.5 cm, and were visually easily distinguishable from each other. The overall envelope (corresponding to contour length in Rousselle et al. [18]) of a set was kept constant at 9 × 9 cm, as overall envelope has been found to help children to make appropriate judgements, even in conditions where overall surface is incongruent with number [10]. The children's task was to find out which set contained more dots, and to respond by pressing the button at the side of the 'winner' set. Response side was counterbalanced.

The size of dots was constant within set and varied between sets. The individual size of dots and the pattern of dots were varied randomly through pairs of sets. Sets with the same number of items never had the same dot size. Only numerosities above the subitization range were used in order to exclude object-based attention responding (based on the object file system, [see [58-61]].

The following factors were varied systematically for the purposes of analysis: (1) The ratio of the number of dots in the two sets. (2) The type of the physical control variable. (3) The congruity of physical control variables and numerosity. We also attempted to control for both problem size (the total number of items) and for numerical distance (the numerical difference between the two sets) in order to avoid that either of these parameters would distort the ratio effect, as ratio effect is the composite of numerical distance and size. The ratios and numerical distances for all combinations of numerosities are summarized in Table 1.

**Table 1:** The dot number pairs per each ratio.

1 :	2	3 :	5	2 :	3
4	8 *	6	10 *	8	12 *
6	12 **	9	15 **	12	18 **^/^°
10	20°	12	20°		

The numerical distance between dot numbers are the same in dot number pairs marked with * and ** (the numerical distances are 4 and 6, respectively). Ratios are in columns. The overall sum is almost equal for dot number pairs marked with ° (30, 32 and 30).

There was no extreme large numerical distance for the 2:3 ratio because the sum of items would have been much larger than in other conditions (at least 16:24, sum is 40; or 20:30, sum is 50). Rather, we decided to keep the sum of items for the largest numerical distances in the 1:2 and 3:5 ratio conditions to be approximately equal to the sum of items in the distance 6 condition in the 2:3 ratio condition.

Two different physical variables of the dot groupings were manipulated as perceptual controls: overall surface (hence, luminance) and overall circumference (sum of the individual items' circumferences). These perceptual controls were intermixed during stimulus presentation. The ratios of the overall physical sizes (surface in half of the trials, and circumference in the other half of the trials) of the dot sets were congruent or incongruent with the numerical ratio of the dot sets. In the congruent condition, the more numerous set was larger in overall physical size than the less numerous set. In the incongruent condition, the more numerous set was smaller in overall physical size then the less numerous set. Congruent and incongruent trials were pseudo-randomly intermixed (no more than three of each could occur consecutively in a sequence).

In each trial the ratio of perceptual features of the two dot patterns was kept the same as their numerical ratio. This was done to ensure that the influence of perceptual variables did not differ for different numerical ratios. For example, if the ratio between the numbers was 1:2 and the ratio between perceptual variables was 2:3, the perceptual difference would be less salient than the numerical difference. This would result in better numerical discrimination performance solely because the perceptual variables would be less distracting. Similarly, if the perceptual ratio were 1:2 and the numerical ratio were 2:3, the perceptual difference would be more salient.

Each child was presented with 64 test stimuli pairs, preceded by 8 practice pairs. Trials were separated by funny smiley figures and friendly pictures of animals and soft toys, to retain children's attention and motivation. Encouraging verbal feedback was given after each trial, independently of performance. Trials began when children attended to the screen and indicated that they were ready for the next task. All children enjoyed the tasks and actively sought participation.

The exceptionally large number of trials allowed for a full factorial ANOVA. We also measured high-precision reaction time. To our knowledge, RT has never been analyzed (or reported) in previous studies of magnitude discrimination with children of this age. Information from RT analyses may offer significant advantages in understanding cognitive processing in children, for example serving as a complementary source of information (in fact, RT was found to be more informative than accuracy in school children's magnitude comparison performance [15,62]).

## Results

### Behavioural tests

A multiple analysis of variance (MANOVA) was performed on the 12 different behavioural tests (number knowledge (recitation and recognition), verbal fluency (animals and colours), verbal short term memory (numbers and words), counting abilities ("Count as far as you can", "How many toys are there" and "Give me a number"), problem solving abilities (easy and difficult), and line halving). Raw scores on each task were used as dependent variables with Age and Gender as independent variables. Results from the twelve tests were entered into univariate F-tests. A Bonferroni-type adjustment was performed in order to lower the possibility of an inflated type I error due to multiple comparisons. The critical alpha-level set by the Bonferroni-type adjustment was 0.0042 (0.05/12). For further analysis of significant MANOVA and F-test results, post-hoc Scheffé tests were implemented.

**Figure 2:**
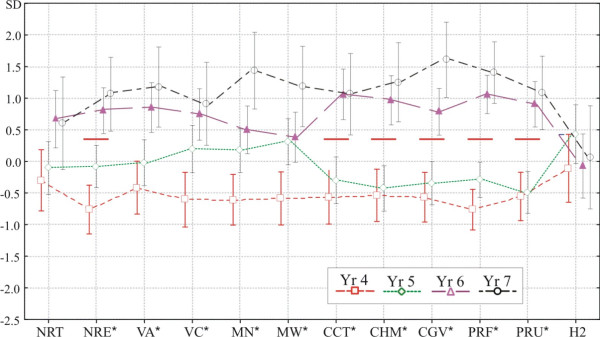
**Standardized scores (units in SD) for the 12 tests separately for each age group**. The critical p value for significance was 0.0042 (see text and Table 2). See text and Table 2 for abbreviations of tests. Significant age group differences are denoted by red lines. See text for more details.

Normalized test results are plotted in Figure 2. The data were normalized so that all tests could be visualised on a common scale, independently of the metrical differences between the different tests. The MANOVA demonstrated that age was a significant factor (Wilks'Ë = 0.11, F(48,175.38) = 2.83, p < 0.0001). The results of follow-up univariate ANOVAs are given in Table 2. The effect of Age was significant in 10 of the 12 tests. Post-hoc Scheffé tests showed that in 6 out of 10 tests (number recognition, counting abilities and in verbal arithmetic abilities) there was an apparent developmental change between the ages of 5 and 6 years: children aged 4 and 5 did not differ from each other significantly; and neither did children between 6 and 7. However, the difference between these two age ranges was significant: both 6- and 7-year-olds performed better than any age group in the younger range.

**Table 2:** Univariate F-tests for the 12 tests.

Task	Age		Gender	
	
	F(4, 56)	p	F(1, 56)	p
**Number knowledge**				
- **NRT: **Say as many numbers as you can	3.18	0.0199	0.61	0.4
- *NRE: Written (Arabic) number recognition	14.58	***<0.0001***	0.91	0.3
**Verbal knowledge**				
- *VA: Say as many animals as you can	8.66	***<0.0001***	4.7	0.033
- *VC: Say as many colours as you can	9.53	***<0.0001***	0.8	0.4
**Working memory (verbal)**				
- *MN: Short term memory for numbers	10.7	***<0.0001***	3.12	0.08
- *MW: Short term memory for words	8.19	***<0.0001***	4.78	0.033
**Counting abilities**				
- *CCT: Count as far as you can	12.54	***<0.0001***	1.1	0.3
- *CHM: How many objects are there	13.82	***<0.0001***	0.8	0.8
- *CGV: Give me a number	15.37	***<0.0001***	0.9	0.3
**Verbal counting abilities**				
- *PR1: Problems - simple	28.85	***<0.0001***	2.1	0.2
- *PR2: Problems - difficult	14.88	***<0.0001***	2.9	0.09
**Fractions**				
- **H2: **Halving	1.23	0.3	0.04	0.8

The critical p value for significance was 0.0042 (set by the Bonferroni adjustment). Significant p levels are denoted by bold italic typesetting.

### Magnitude comparison task

As accuracy data is binary in nature, summarized as proportions, the arcsine transformation was applied to the data. Statistical analyses were carried out on both raw proportions and on arcsine-transformed accuracy data. As the results were mostly identical, we report p-values from the transformed data in square brackets only when the results were slightly different. Figures show row accuracy proportions for the sake of intelligibility.

**Figure 3:**
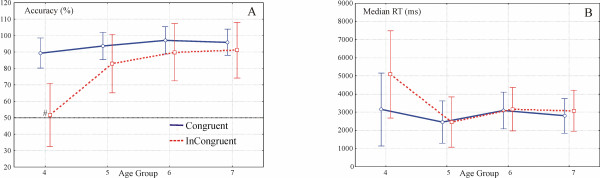
**Congruency effect**. A. Congruency effect in accuracy across age groups. One-sample t-tests showed that performance in the Incongruent condition did not differ from chance in this age group (the non-significant one-sample t-test is denoted by #). B. Significant Age × Congruency interaction in RT (p < 0.02).

First, in order to see whether accuracy was significantly different from chance at the group level, one-sample t-tests were run against 50%. Second, accuracy and median reaction time (RT) were calculated and entered into ANOVAs, taking Side (of the response) × Congruency × Type (of perceptual control) × Ratio as within-subject factors. Age and Gender were the between-subject factors. The main effect of **Age **was highly significant with respect to accuracy (Figure 3, F(1,58) = 11.9, p < 0.0001).

Post-hoc Scheffé tests indicated that the youngest group made significantly more errors than the other three age groups in the Incongruent condition (p < 0.001). There were no significant differences among the three older groups (accuracy and RT data is shown in Table 3). The main effect of **Gender **was marginally significant for accuracy (F(1, 58) = 3.8, p = 0.057 [p = 0.062]). Boys committed fewer errors than did girls (89.4% vs. 83.5%). Neither of the above main effects was significant in RT.

**Table 3:** Performance in the magnitude comparison task.

Group	Accuracy (%)	Median RT (ms)
4 yrs	70.5 (3.26)	4123 (586.2)
5 yrs	88.3 (3)	2465 (338.5)
6 yrs	93.4 (2.96)	3135 (291.7)
7 yrs	93.6 (2.87)	2941 (276.4)

Standard errors are given in brackets.

The main effects of **Congruency **(Accuracy: F(1,58) = 31.8, p < 0.0001; RT: F(1,58) = 11, p < 0.002) and **Ratio **(Accuracy: F(1,58) = 23.4, p < 0.0001. RT: F(1,58) = 22.5, p < 0.0001) were highly significant. Children responded faster in congruent trials and with fewer errors than in incongruent trials (2880 vs. 3452 ms, 94% vs. 78.9%). More-different ratio pairs were responded to more accurately and faster than less-different ratio pairs (2422, 3062, 4014 ms and 90.8%, 85.5%, 83.2% correct for Ratios 1:2, 3:5 and 2:3, respectively). The **Type **of perceptual control stimulus was not significant for accuracy nor RT (p > 0.4).

The **Age × Congruency **interaction was significant both in accuracy (Figure 3/A, F(3,58) = 7.5, p < 0.001) and in RT (Figure 3/B, F(3,58) = 3.96, p < 0.02). Pairwise post-hoc comparisons revealed that the effect of Congruency on accuracy decreased with increasing age, reaching statistical significance only in the youngest group (p < 0.001). One-sample t-tests confirmed that all age groups performed significantly above chance in both Congruent and Incongruent conditions except for the 4-year-old group, whose performance did not differ from chance in the Incongruent condition (subsequently for Ratios 1:2, 3:5 and 2:3: 59, 50.5 and 45.5% correct. T-test results in the same order: t(13) = 0.99, p > 0.3, t(13) = 0, p = 1 and (13) = -0.42, p > 0.6) Post-hoc tests yielded non-significant results for RT data (p > 0.1).

The **Gender × Congruency **interaction was also significant (Accuracy: F(3,58) = 5.36; p < 0.03. RT: F(3,58) = 5.9; p < 0.03). Girls' accuracy was significantly affected by Congruency (94.1% vs. 72.9% in congruent and incongruent trials respectively, p < 0.0002). In contrast, the Congruency effect was not significant for boys (93.8% and 84.6%, p > 0.16). No similar effect was found in RT data (p > 0.1). According to post-hoc comparisons, girls' performance was significantly worse in the incongruent condition than boys' (p < 0.04 [p = 0.065]). In contrast, the performance of boys and girls did not differ in the congruent condition (p > 0.99). The three-way interaction of Congruency, Gender and Age was not significant (p > 0.3) indicating that the gender difference in the congruency effect is stable across age groups. No such result emerged from the RT data.

**Figure 4:**
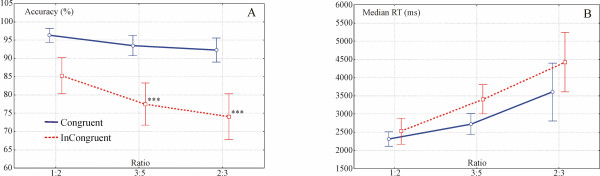
**Congruency × Ratio interaction**. A. Congruency × Ratio interaction in accuracy. Ratio effect was significant only in the Incongruent condition (*** denotes p < 0.001 significance level). B. The same interaction was marginally significant in RT data (p = 0.066).

The **Congruency × Ratio **interaction was significant in accuracy (Figure 4/A): F(2, 116) = 4.7; p < 0.02) and it was marginally significant in RT (Figure 4/B, p = 0.066).

Post-hoc tests revealed that there was a steeper Ratio effect in the accuracy data in the incongruent trials (p < 0.0005 between ratio 1:2 and ratio 3:5; and p < 0.0002 between ratio 1:2 and ratio 2:3), than in the congruent trials. Incongruent trials yielded more mistakes in the more difficult ratios. In RT, planned comparisons revealed that the ratio effect was significant in both congruent and incongruent conditions (p < 0.001 between ratio 1:2 and 3:5; and between ratio 2:3 and 3:5).

Interestingly, the **Age × Gender **interaction was significant in RT (F(3,35) = 3.3, p = 0.029) but not in accuracy. Boys responded faster than girls in the 4-year-old group (2495 vs. 5750 ms, the difference is 3254 ms). This large discrepancy was not present in the older groups (2605 vs. 2325 ms, 3558 vs. 2712 ms, and 2859 vs. 3023 ms, respectively). None of the post-hoc comparisons was significant.

### Correlations and factor analysis

In order to investigate the relationship of counting abilities and magnitude comparison, correlational analyses were carried out. Variables were the outcomes of the behavioural tests, and median RT and accuracy for each condition of the magnitude comparison task (3 levels of Ratio and 2 levels of Congruency). Further, derived variables such as the slope of the Ratio effect (RT and accuracy for smaller ratio minus RT and accuracy for larger ratio), and Ratio × Congruency cells (accuracy and RT data for each Ratio within each Congruency condition) were also used for analysis. Again, both raw accuracy proportions and arcsine transformed accuracy data were entered into the analyses. P-values of the arcsine transformed data are denoted by superscripts when different from the p-values of the raw data. Age was controlled by computing partial correlations. Further, the 4 age-groups were also analysed separately, in order to reveal any possible group differences in correlations.

The matrix of correlation coefficients (controlling for age) for test results is shown in Table 4. The correlation coefficient matrix for the magnitude task results is shown in Table 5.

**Table 4:** Partial correlations among tests.

	NRT	NRE	VA	VC	MN	MW	CCT	CHM	CGV	PR1	PR2	H2
**NRT**												
**NRE**	**0.27**											
**VA**	0.20	0.10										
**VC**	0.24	**0.35**	**0.39**									
**MN**	0.22	0.19	0.24	0.12								
**MW**	0.16	**0.28**	0.21	0.16	**0.46**							
**CCT**	**0.37**	0.25	**0.33**	0.08	0.12	-0.08						
**CHM**	**0.31**	**0.26**	**0.34**	-0.01	0.21	0.00	**0.82**					
**CGV**	0.21	0.17	**0.29**	0.00	0.18	0.04	**0.77**	**0.84**				
**PR1**	0.13	0.14	**0.40**	0.20	0.11	-0.02	**0.45**	**0.37**	**0.28**			
**PR2**	**0.33**	0.07	**0.48**	0.05	0.15	0.10	**0.46**	**0.56**	**0.47**	**0.51**		
**H2**	-0.04	-0.06	-0.05	0.04	-0.09	0.00	-0.13	-0.16	-0.14	**-0.29**	-0.19	

R values from partial correlations for tests. Age was controlled. Bold typesetting indicates significant (p < 0.05) correlations. **NRT: **Say as many numbers as you can; **NRE: **Written (Arabic) number recognition; **VA: **Say as many animals as you can; **VC: **Say as many colours as you can; **MN: **Short term memory for numbers; **MW: **Short term memory for words; **CCT: **Count as far as you can; **CHM: **How many objects are there; **CGV: **Give me a number; **PR1: **Problems - simple; **PR2: **Problems - difficult; **H2: **Halving

**Table 5:** Partial correlations among magnitude task measurements.

		SPEED (RT)						ACCURACY (%)				
		CON	NCON	RAT1	RAT2	RAT3	ALL	CON	INCON	RAT1	RAT2	RAT3
**SPEED (RT)**	**CON**											
	**INCON**	**0.54**										
	**RAT 1**	**0.71**	**0.68**									
	**RAT 2**	**0.83**	**0.68**	**0.68**								
	**RAT 3**	**0.85**	**0.63**	**0.63**	**0.68**							
	**ALL**	**0.92**	**0.74**	**0.80**	**0.88**	**0.93**						
											
**ACCURACY**	**CON**	**-0.26**	0.00	**-0.33**	**-0.32**	0.00	-0.18					
	**INCON**	-0.14	**-0.27**	**-0.32**	**-0.26**	-0.06	-0.19	**0.25**				
	**RAT 1**	-0.18	-0.21	**-0.27**	**-0.35**	0.02	-0.18	**0.54**	**0.85**			
	**RAT 2**	-0.17	-0.22	**-0.38**	**-0.28**	-0.04	-0.20	**0.52**	**0.89**	**0.81**		
	**RAT 3**	-0.24	-0.22	**-0.42**	**-0.32**	-0.10	-0.25	**0.57**	**0.86**	**0.76**	**0.81**	
	**ALL**	-0.21	-0.23	**-0.39**	**-0.34**	-0.05	-0.22	**0.58**	**0.93**	**0.91**	**0.94**	**0.93**

Age was controlled. Bold typesetting indicates significant (p < 0.05) correlations. [Arcsine transformed data: Asterix indicates correlations where p = 0.07. Circle indicates where p > 0.1].

As it can be seen in these correlation matrices, there are strong correlations among some number knowledge, verbal fluency, counting and problem solving tasks; among reaction time measurements; and among accuracy measurements. In order to draw a concise summary of these interrelations, we performed a factor analysis. The factor analysis would also show clearly that which abilities form together a common factor and which abilities are independent of each other. The factor analysis (controlling for age) confirmed the above intercorrelations and yielded the following factors: 1) 'Counting, number knowledge and verbal fluency'; 2) 'Speed'; and 3) 'Accuracy'. Table 6 summarizes the results of the factor analysis.

**Table 6:** Factor analysis.

	1	2	3
**Tests**			

**NRT**	0.239429	0.539796	0.117110
**NRE**	0.190808	**0.705857**	0.145507
**VA**	0.050910	**0.712302**	0.258825
**VC**	0.120982	0.557922	0.268724
**MN**	0.175689	0.551612	0.379478
**MW**	0.170932	0.405931	0.409953
**CCT**	0.049560	**0.909566**	0.164922
**CHM**	0.036905	**0.889526**	0.213583
**CGV**	0.044615	**0.867434**	0.223804
**PR1**	0.058252	**0.824896**	0.223152
**PR2**	0.143934	**0.807914**	0.217954
**H2**	-0.022292	-0.101818	-0.107875

**Comparison - speed**			

**CONGRUNENT**	**-0.930375**	-0.093792	-0.103606
**INCONGRUENT**	**-0.722560**	-0.013829	-0.208961
**RATIO 1**	**-0.724875**	-0.188580	-0.400200
**RATIO 2**	**-0.855544**	-0.115420	-0.317704
**RATIO 3**	**-0.924577**	0.053293	0.126029
**ALL**	**-0.982134**	-0.056095	-0.133725

**Comparison - accuracy**			

**CONGRUENT**	0.173563	0.117307	0.640845
**INCONGRUENT**	0.131973	0.220069	**0.892832**
**RATIO 1**	0.128044	0.162317	**0.909502**
**RATIO 2**	0.143302	0.232862	**0.911971**
**RATIO 3**	0.195263	0.224094	**0.892648**
**ALL**	0.167608	0.221621	**0.954582**

**Expl. Var.**	9.784019	6.247673	5.951855

Factor loadings. Extraction method: principal components were extracted and varimax rotation was applied. Marked loadings are > 0.7.

Consequently, global indices of the three factors were calculated, by averaging the performance values of the measures contributing to each factor. 'Speed' and 'Accuracy' were then correlated with behavioural tests, and 'Counting, number knowledge and verbal fluency' was correlated with performance in the magnitude comparison task. 'Accuracy' significantly correlated with the two memory measures (memory for numbers: r = 0.246, p = 0.05; and memory for words: r = 0.29, p < 0.03). No other correlations were significant.

In order to investigate the unique variance explained by the short term memory measures in 'Accuracy', a regression analysis was carried out with 'Accuracy' as the dependent variable (DV) and *short term memory for numbers *and *short term memory for words *as independent (predictor) variables (IVs). The regression model was significant and the two short term memory measures accounted for 21% of the variance in 'Accuracy' (adjusted R^2 ^= 0.211; F(2,63) = 9.7, p < 0.001). Neither of the memory tests accounted for a significant amount of variance on its own (but both showed a strong trend: memory for numbers: t = 1.76, p = 0.08; and memory for words: t = 1.95, p = 0.06).

## Discussion

In this study, we set out to explore the developmental relations between magnitude representation, number knowledge and counting skills in children aged 4 to 7 years. In order to get around some unwanted confounds, we made a number of methodological innovations to the basic number comparison task.

In response to our main developmental question, we found no relationship between non-symbolic number comparison performance and number or counting knowledge. These data are consistent with recent studies reporting no relationship between non-symbolic magnitude comparison and arithmetic performance in 6-8 year-old primary school children [62] and in second, third and fourth graders with mathematical disabilities [63,64]. In addition, performance measures on the non-symbolic number comparison task ('Speed' and 'Accuracy') and performance on number/counting knowledge tasks ('Counting, number knowledge and verbal fluency') form factors which are independent of each other. Again, this suggests that non-symbolic number comparison skills and symbolic counting/number knowledge develop in isolation between 4-8 years of age. However, we cannot exclude that there may be a relationship between non-symbolic number comparison and counting knowledge before 4 years of age [17,18].

Regarding the non-symbolic number comparison task, a significant congruency effect was found. The Congruency effect was of interest because it can attest whether children attended to numerosity or to irrelevant perceptual variables. We found that 4 year-olds are not able to perform intentional numerical judgments independently of physical appearance: their performance was at chance when the perceptual information was in conflict with numerical information. Susceptibility to irrelevant perceptual features weakened with age, and the congruency effect was mainly driven by 4-year-olds. This developmental trend is in agreement with previous studies [16,18,19]. Although Halberda and Feigenson [16] reported above-chance performance when the data for all ratios were collapsed, their Figure 2 reveals that 3- to 4-year-old children were at chance with more difficult ratios. The detailed analysis of possible ratio × congruency interaction is worthwhile: the main effect of congruency might be distorted by the data from conditions when the comparison is easier.

Our finding that 4-year-old girls performed more poorly than boys needs to be replicated. At this point, we assume that gender differences were due to differential familiarity with computers and computer games between the genders.

Performance in the incongruent condition showed developmental progression between 4 and 5 years of age. This change was independent of and preceded the developmental change in verbal and counting abilities and simple arithmetic, which occurred between 5 and 6 years of age (this latter is most probably due to start of systematic preparation for school at this ages in Hungarian kindergartens). The lack of correlation between congruency effects and counting abilities and simple arithmetic suggests that children's sensitivity to incongruent irrelevant stimulus dimensions was independent from counting and arithmetic knowledge. A non-numerical explanation of these results might be that older children had more mature cognitive and motor inhibition capacities than younger children and that this contributed to the more efficient processing of task-relevant information in general. For example, there is ample evidence that older children perform better in a range of Stroop-like tasks than younger children [16,65,66]. Indeed adult level performance is not reached on Stroop tasks till the age of 21 [67]. Moreover, it has been shown that automatic processing of irrelevant physical properties is inevitable even in adults and that these properties exert a significant effect even on adult's performance in dot comparison [68]. Similarly, we ourselves have shown that 5.5 to 9-year-old primary school children and adults demonstrate substantial incorrect motor activation in the incongruent conditions of symbolic and non-symbolic Stroop tasks [69,70]. Overall, the evidence suggests that general conflict resolution skills, most probably cognitive and motor inhibition abilities, substantially contribute to performance in judgement tasks such as those used here, where stimuli have both task-relevant and task-irrelevant properties.

It is also worth noting that the 'Accuracy' factor in magnitude comparison correlated with performance on short term memory tasks in our study. This indicates that general abilities like memory may play an important role in children's performance in non-symbolic number comparison. Accordingly, some studies have identified working memory and executive functions as significant factors in arithmetic development [45-47,51,52]. We do not yet have a strong basis for any firm conclusions on the role of short term memory in number comparison. Probably, better memory abilities help children to keep the task-relevant dimension in their minds so that they can ignore the task-irrelevant features easier. The possibility of more complex causal relations among magnitude representation, symbolic maths and general abilities (inhibition, memory, attention) should be considered in any further research into the development of magnitude representation and arithmetic performance. For example, longitudinal studies have shown that cognitive-linguistic skills and also motivational factors (such as non-verbal intelligence, preschool counting skills, task orientation and social dependence) predict later maths performance during school years [71,72].

It is also interesting to speculate whether the shifting relationship between non-symbolic and symbolic magnitude comparison performance and maths performance reflects a shifting landscape of developmentally singular events. For example, correlation of non-symbolic magnitude comparison performance and maths skills at age 3-4, but not later, may point to the importance of establishing numerical abstraction at this particular age [17,18,39,41,42]. Similarly, the correlation between maths performance and symbolic number comparison skills at age 6-8 [15,62] may illustrate the importance of establishing links between symbolic numbers and referents at this particular age. The developmental challenge would then be to identify age-appropriate markers which reflect the most significant achievements of children at particular ages. Most probably, these markers will change continuously during development, depending on the most significant learning events in children's lives. This notion is similar to Siegler's overlapping waves theory [73], which suggests that the relative dominance of particular strategies change continuously during development. Similarly, the relative importance of developmental markers probably also shifts in a continuous manner. That is, while the symbolic distance effect may be a good predictor of arithmetic development between ages 6-8, this relationship may weaken later (Ansari, personal communication).

To illustrate how optimal developmental markers may change with age and experience, we can draw an analogy with musical cognition. At the beginning of learning to play the violin, for example, it is interesting to know whether better discrimination between violin strings correlates with better musical abilities. In fact, playing each note is directly related to the ability to discriminate between the strings and string positions. Hence, violin string discrimination is a necessary causal precondition of playing violin music. However, later in musical development it is likely that the more music children have played during the past 10 years, the better they discriminate violin strings. At this point, better string discrimination becomes a relatively unimportant consequence of playing more music. Accordingly, at this developmental time point, it is unlikely that complex musical skills are related to violin string discrimination. In other words, after a violin string-musical note connection has been established, practice effects on string discrimination ability will no longer capture the most important developmental markers (these may now be the understanding of tempo, melody, interpretation, etc., related to much higher-level musical concepts).

The argument is similar for maths and predictors of school maths abilities. For example, a recent study reported a correlation between non-symbolic dot comparison performance measured at age 14 and maths performance measured during 5-11 years of age [74]. As causal directions cannot be determined by correlative studies, the significance of these results with regard to representational development is not clear. While the authors argued that the most likely explanation for their data was that the development of non-symbolic magnitude comparison enhanced arithmetic performance, it is equally likely that better counting/arithmetic skills, and/or more experience with numbers, resulted in better set estimation skills [75].

## Limitations

Some limitations should be noted before we draw the final conclusions. First, we did not have children younger than 4 years of age in our study. The picture of the relationship and co-development of verbal abilities and magnitude representation would be complete only with the examination of 2- and 3-year old children. Second, more tests measuring specifically working memory and executive functioning would have been necessary for this study to make firm conclusions of the role of these abilities in numerical development.

## Conclusions

This study examined the proposed relationship among verbal counting knowledge, arithmetic performance and non-symbolic magnitude representation. We found that verbal counting knowledge and performance on simple arithmetic tests did not correlate with non-symbolic magnitude comparison at any age. Regarding the non-symbolic magnitude representation, it was found that older children (5+ years) performed successfully on the number comparison task, showing behavioural patterns consistent with an analogue magnitude representation. In contrast, 4-year-olds were unable to discriminate number independently of task-irrelevant perceptual variables. Sensitivity to irrelevant perceptual features of the magnitude discrimination task was also affected by age, and correlated with memory, suggesting that more general cognitive abilities may play a role in performance in magnitude comparison tasks. We conclude that young children may not able to discriminate numerical magnitudes when co-varying physical magnitudes are methodically pitted against number. Under these conditions, executive functioning, especially inhibition may play the most important role. Verbal counting knowledge and non-symbolic magnitude comparison abilities were independent of each other in 4-7-year-old children, as shown by the factor analysis.

## Competing interests

The authors declare that they have no competing interests.

## Authors' contributions

FS took part in the planning and designing the experiment and behavioral tests, performed the data analyses and drafted the manuscript. DS contributed to the design and planning of experiment and behavioural tests, and helped drafting the manuscript. LS took part in the planning of behavioral tests and collected all the data. All authors read and approved the final manuscript.
